# Associating maternal keel fracture severities with egg quality, hen reproductive outcome, and chick welfare

**DOI:** 10.1016/j.psj.2025.105894

**Published:** 2025-09-23

**Authors:** M.O. Logunleko, S.L. Lambton, G.J. Richards, J.L. Edgar

**Affiliations:** a Bristol Veterinary School, University of Bristol, Langford BS40 5DU, UK; b Department of Animal Physiology, Federal University of Agriculture, Abeokuta, PMB 2240, Nigeria

**Keywords:** Keel bone, Chicken, Welfare, Maternal effect, Bone damage

## Abstract

Maternal conditions are known to affect offspring behavior and performance in laying hens. With evidence suggesting that keel fractures cause pain and may lead to stress and fear in affected hens, this study hypothesized decreased egg quality, reproductive outcomes, offspring chicks’ weight, and increased fear responses in the chicks as keel fracture severity increased.

120 Bovans brown layer breeders aged 61 weeks, with different keel fracture severities, were co-housed with 12 cocks at 20 hens+2 cocks/room. Eggs laid between weeks 65-70 were collected either for incubation or egg quality assessments. At week 70, 111 hens were euthanized, keel bones were dissected and scored (0:no fracture, *n* = 21; 1:slight fracture, *n* = 25; 2:moderate fracture, *n* = 29; 3:severe fracture, *n* = 36). Majority of the fractured hens (88 out of 90) presented hard callus without fracture gaps, suggesting healed fracture status. At hatch, 612 chicks were generated. Chicks’ body weight was measured weekly until week 5. All chicks were subjected to tonic immobility (week four) and novel arena tests (week five).

Dry shell weight was lower for moderate (*P* = 0.007) and severely (*P* < 0.001) fractured hens, while eggshell index was lower in severely fractured hens (*P* < 0.001) compared with those without fractures. Shell thickness decreased as fracture severity increased (*P* < 0.001). Overall, egg-breaking force was lower (*P* < 0.001) among fractured hens, but there was no difference in breaking force of the three fracture severity groups. Lower hatch rate was recorded in moderate (*P* = 0.038) and severely (*P* = 0.003) fractured hens compared with those without fractures. Irrespective of severity, chicks from fractured hens had lower odds (*P* < 0.05) of performing escape attempts, higher likelihoods (*P* < 0.001) of freezing and sitting inactive.

In summary, layer breeders with moderate and severe fractures, even after healing, produced eggs with lower shell quality, breaking strength, and hatch rate. At higher prevalence, this may have implications for the managerial practice and economic return of a breeder flock. Maternal keel fracture was associated with altered fear behavioral patterns in chicks, with a shift from active to passive responses.

## Introduction

Globally, the laying hen industry remains an important source of protein (Castro et al., 2023; Medina-Cruz et al., 2024). In recent decades, consumers have been demanding good rearing systems and conditions (indicative of good welfare) for chickens (Marian and Thøgersen, 2013; Sismanoglou and Tzimitra-Kalogianni, 2019; Institute, 2020; Cao et al., 2021). Thus, good welfare ranks alongside production priorities for the industry. However, keel bone fractures remain a major welfare concern for laying hens. Keel fractures have been reported at higher prevalence in several studies (Rodenburg et al., 2008; Wilkins et al., 2011; Petrik et al., 2015; Thøfner et al., 2021), although prevalence varies depending on combinations of different risk factors, such as housing design (Wilkins et al., 2011; Casey-Trott et al., 2017; Eusemann et al., 2018; Hardin et al., 2019), breed (Candelotto et al., 2017), laying efficiency (Gebhardt-Henrich and Frohlich, 2015) and bird age (Casey-Trott et al., 2017; Wei et al., 2020, 2022). Furthermore, the occurrence of keel fracture has been negatively associated with measures of welfare, such as reductions in motivated behaviors, like perching (Nasr et al., 2012a; Wei et al., 2020), locomotion (Rentsch et al., 2019; Montalcini et al., 2024), as well as a reduction in egg production and quality (Nasr et al., 2012a; 2013; Wei et al., 2020; Rufener et al., 2019). Evidence suggests that keel fractures are associated with behavioral indicators of pain in 35-week-old hens (Nasr et al., 2012b) and high levels of fearfulness both at 42 and 56 weeks of age (Zhang et al., 2022; Wei et al., 2020). A keel fracture could induce chronic stress in affected chickens (Armstrong et al., 2020).

Efforts to understand the consequences of keel fractures have focused on the injured birds themselves. However, parental effects are recognized as influencing the life trajectory of birds (Janczak et al., 2007). Such parental effects could be in the form of maternal stress arising from environmental conditions, which include diet, light, temperature, pathogens, social conditions, management, and housing conditions (de Haas et al., 2021). In breeder hens, positive and negative environmental factors (Angove and Forder, 2020), as well as stressors experienced during the reproductive stage (Peixoto et al., 2020), influenced the growth and welfare of offspring chicks**.** For example, restricted feeding of breeder hens resulted in elevated tonic immobility and low feeding in a competitive feeding test among their offspring chicks (Janczak et al., 2007).

Sensitive periods for maternal effects include both egg formation and embryogenesis (de Haas et al., 2021). There are several mechanisms through which maternal conditions could influence the programming of developing embryos. One such mechanism is through epigenetics (Hynd et al., 2016). The epigenetic mechanism can be induced by stress and/or the environment (Goerlich et al., 2012; Jensen, 2013; Dunislawska et al., 2022). According to Jensen (2013), animals’ experiences could mediate DNA and histone changes, which may result in behavioral modifications. Such epigenetic modifications can be transferred across generations (Jablonka and Raz, 2009; Natt et al., 2009; Jensen, 2013). There is evidence of transgenerational transfer of epigenetic behavioral alteration in chickens. Two studies exposed hens to a chronic unpredictable light rhythm that lasted from the rearing to the laying phase. Study 1 (Natt et al., 2009) reported a modification in brain gene expression and greater foraging behavior in female offspring. Similarly, the offspring of stressed hens in study 2 demonstrated reduced spatial learning ability coupled with higher feed competitiveness compared with the offspring of unstressed parents (Lindqvist et al., 2007). Another study by Goerlich et al. (2012) reported that when hens were subjected to 3 weeks of intermittent social isolation post-hatch, their male offspring, compared with the control, had a suppressed corticosterone response to restraint in a similar way to their mother. Findings from these studies validate the epigenetic effect of maternal condition in chickens.

Alternatively, maternal stress experience could be signaled to the developing embryo either through hormone deposition in the egg (Peixoto et al., 2021) or through changes in yolk composition, as a way of programming adaptive responses to changing environments (Gluckman et al., 2005; Hynd et al., 2016). The egg deposition of maternal nutrients, immune factors, and hormones could affect the behavior, morphology, and physiology of the offspring (Groothuis and Schwabl, 2008; Ho and Burggren, 2010). A study by de Haas and colleagues demonstrated that high maternal plasma corticosterone in Dekalb White was associated with increased occurrence of injurious feather pecking in offspring (de Haas et al., 2014). Excess circulation of maternal plasma corticosterone may be transferred into an egg, as supported by a study where plasma elevation of corticosterone lowered yolk concentration of steroid hormones (Henriksen et al., 2011). Offspring from the hens with plasma-elevated corticosterone in a follow-up study had lower hatch weight, with reduced fear level and competitiveness (Henriksen et al., 2013). In a different study, exposure of hens to unpredictable human presence for 1 minute daily, five times a week, over five weeks, resulted in lower yolk progesterone and estradiol concentration (Bertin et al., 2019). The study also recorded a reduction in the offspring chicks' ability to differentiate unfamiliar from familiar conspecifics in a social discriminant test. Therefore, if keel fractures cause stress in layer breeders, it is possible that this could have significant implications for the behavior and welfare of their offspring, mediated either by epigenetics or deposition of certain factors in the egg.

A third mechanism through which maternal conditions could possibly influence offspring is through changes in egg quality. Changes in egg and shell properties may affect embryonic mortality and hatchability (Shafey, 2002). Calcium is an important mineral for eggshell formation (Riber et al., 2018; Jin et al., 2024), with a complete egg containing around 3 g of calcium (Robert, 2004). The shell thickness is said to be a source of calcium for developing embryos (Nys et al., 2004), with chicks reportedly obtaining about 80 % of their required skeletal calcium from the mammillary layer of the eggshell during incubation (Simkiss, 1961; Tabler, 2019). Fractured bones in the hens also require calcium for rapid healing (Einhorn and Gerstenfeld, 2015; Fischer et al., 2018). Thus, with competition for calcium between eggshell formation and bone formation, there is the possibility of calcium redistribution in fractured hens in favor of healing (Scholz et al., 2008), which may reduce egg and shell qualities. Indeed, reports from previous research emphasized how recent keel bone damage resulted in poor egg weight, egg strength, and eggshell thickness between 22 and 42 weeks of age (Nasr et al., 2012a; 2013; Wei et al., 2020). Similarly, reduced eggshell membranes were reported even in hens with healed fractures (Edgar et al., 2023).

In layer breeder hens, both fertility and hatchability are important measures of reproductive outcomes, and good egg quality is an essential factor for chick hatch quality. According to Angove and Foder (2020), the egg's physical characteristics influence embryonic development and successful hatching. Physical characteristics of high importance include egg weight, eggshell quality, and strength (King’ori, 2011; Kibala et al., 2018; Rayan et al., 2020). Moderately thick eggshell and egg size are essential to ensure embryonic protection from dehydration and pathogens while also promoting gas exchange and nutrient use during incubation (Narushin and Romanov, 2002; Nys et al., 2004). Poor shell quality could result in cracks, thereby reducing the number of hatchable eggs (Nasr et al., 2013; Rayan et al., 2020). Hence, if keel damage adversely affects egg quality, the potential impacts on the efficiency of the layer industry may be significant.

Considering the evidence from above that maternal effects could contribute to early life behavioral development and chick post-hatch performance, coupled with the report that keel bone fractures could induce stress (Armstrong et al., 2020; Rokavec and Šemrov, 2020), elevate blood corticosterone concentration (Wei et al., 2023; 2022) and impair egg quality (Nasr et al., 2012a; Wei et al., 2020), it is possible that the consequences of keel fractures in layer breeders do not end with the injured hens, but extend to their eggs and chicks. This study thereby assessed the possible association between layer breeders’ keel fracture incidence and severity, and their egg quality, fertility, and hatchability, as well as the productivity and welfare of their chicks (as measured using growth and fear metrics). The study hypothesized decreased egg quality, fertility, hatch rate, chick weight, and increased fear responses in chicks as hen fracture severity increases.

## Materials and methods

### Ethical approval

This study was approved by the University of Bristol Ethical Committee (UIN-22-096), and the study was conducted following the guidelines of the Institutional Animal Care and Use Committee **(IACUC)**.

### Experimental animals and housing

120 Bovans Brown breeder hens and 12 cocks were obtained from a breeder farm at 61 weeks of age. Based on the keel bone palpation score (Wilkins et al., 2011), 60 hens had keel fractures, and 60 were suspected to be fracture-free. Hens were then transported to the Agri-Tech Centre UK **(ATCUK)** Poultry Facility at the Bristol Veterinary School in poultry crates, according to DEFRA transport regulations. At the poultry facility, the hens were treated with red mite powder (diatomaceous earth). Each hen was weighed and leg-tagged using combinations of three colors to individually identify them. To avoid room effects, ten hens with keel fractures and ten suspected fracture-free hens were randomly assigned to each of six rooms. Two cocks were randomly allocated to each room to give a mating ratio of 10 hens/cock. The six rooms were in the same building and were identical in dimension (4.2 m x 4.4 m x 2.15 m LxWxH), photoperiod (16L:8D), temperature, relative humidity, ventilation and resources which included litter (5 cm depth wood shavings), two suspended feeders, a 360 cm line of 20 automatic nipple drinkers and two pecking blocks that were placed at similar locations in each room. Hens in each room were provided with two perches (30 cm above the ground; to prevent new fractures) and a 10-unit nest box (2 tiers at 70 cm height above the ground). The chickens had ad libitum access to commercial layer breeder feed and drinking water. They were health checked and allowed to acclimatize in the room for 4 weeks.

### Egg collection procedure for incubation

In weeks 65, 66, and 67, eggs were collected from individual hens on 4 days per week; hence, 3 batches of eggs were obtained (i.e. 1 batch/week). To facilitate individual egg collection, each room had 2 overhead cameras, one centered on the next box and the other covering the entire room. This allowed for 24-hour recordings and remote observation of the hen laying activities. This was combined with live observation by an observer stationed by the entrance of each room during the peak laying hours (8:00 −13:00). When egg-laying behavior (indicated by the hen squatting while stationary and lowering her vent, followed by an instant egg drop), was observed, the observer immediately collected the egg and labelled it according to the room, day and the hen’s color tag. Thus, the mother of each hatched chick could be identified. A total of 1188 eggs were collected across the three batches, and eggs/batch were stored for 3-6 days before incubation.

### Egg incubation

1118 good quality eggs (i.e. without cracks and softshell) were weighed, fumigated with Ambicide disinfectant, and incubated using Brinsea OvaEasy 580 Advance series (Brinsea, USA) at an incubation temperature of 37.5°C, relative humidity of 45 %, and automated hourly turning. Eggs/batch were incubated, ensuring eggs from all rooms were evenly represented across the different setting baskets and chambers of the incubator (Fig. 1a). The eggs were candled on day 18, and the number of fertile eggs/hen as well as the dead germ eggs was recorded. Fertility was calculated as the number of fertile eggs and dead in germ relative to the total egg set. 846 fertile eggs from the 3 batches were transferred into a hatcher (Brinsea OvaEasy 580 Advance Series, Brinsea, USA) at a temperature of 36.5° C and relative humidity of 55 %. Eggs from the same hen were placed in the same row of a mini-chamber, and eggs from all hens were randomly mixed between the mini-chambers and across all levels of the hatcher to avoid a hatcher effect. Each mini chamber was partitioned by doubling the height of the divider to prevent chick mix-up after hatching (Fig. 1b). Between days 21-22 of incubation, 612 chicks (286 male and 326 female) were hatched across the 3 batches derived from 111 dissected hens (see section on hen euthanasia below). The male chicks were killed through cervical dislocation, while the pullets (96, 122, and 108 from batches 1-3, respectively) were transferred to the brooding rooms (see section on chick housing and management below). Hatch rate was calculated as the total number of hatched chicks relative to the fertile eggs that were placed in the hatcher.

**Fig. 1a:**
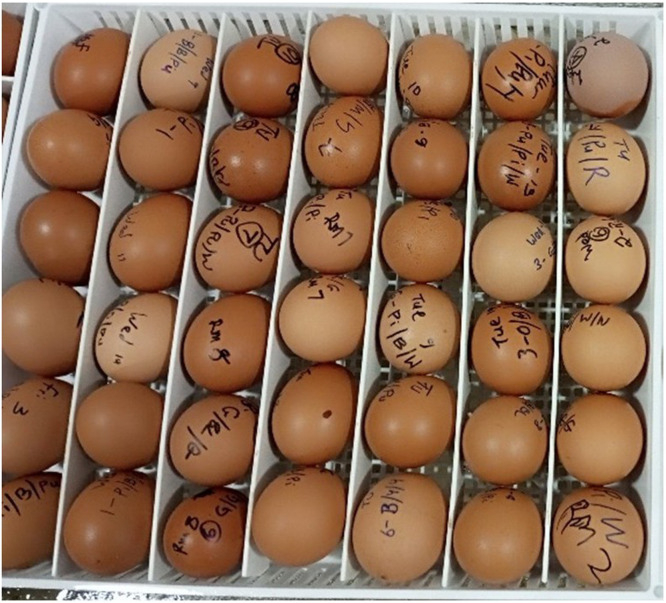
Representation of hens’ eggs across different rooms in the same setting basket.

**Fig. 1b:**
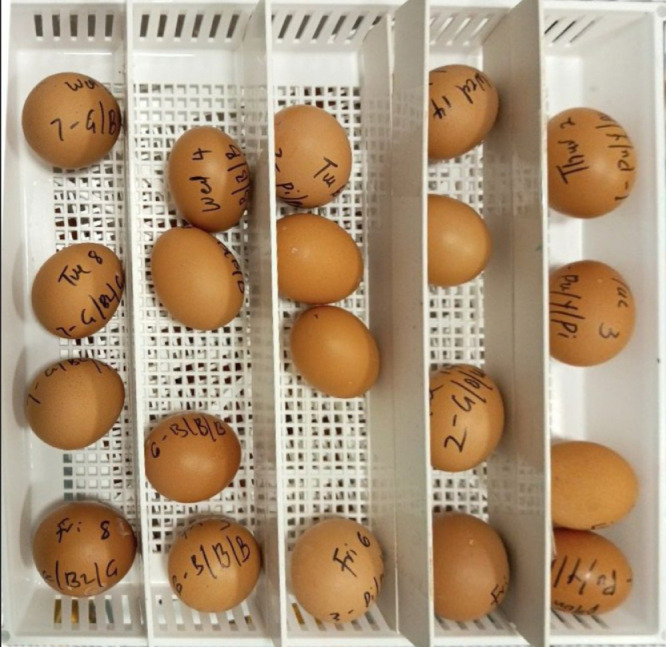
Grouping of eggs/hen with representation of different rooms in the same hatching basket.

### Egg collection procedure for egg quality assessment

In weeks 68, 69, and 70, eggs were collected once a week for three weeks, with individual laying hens identified (as per the egg collection procedure for incubation described above), for egg quality assessment. Each week, 94, 77, and 84 eggs, respectively, were taken to the lab the day after collection for analysis of egg internal and external qualities. Egg quality assessment was done blindly, as the study design relied on the keel dissection score for fracture severity, which was unknown at the time.

### Egg quality assessment

All eggs were weighed (g) using a sensitive measuring scale with an accuracy of 0.01(Mettler PC 4400, Mettler Instrument AG, CH-8606 Greifensee, Zurich).

#### Egg shape index

Egg length and width were measured using a digital caliper with an accuracy of ± 0.001 mm. The egg shape index (%) was calculated according to Das et al. (2010) as;

$$\text{Egg}\text{shape}\text{index}=\frac{\text{Egg}\text{width}}{\text{Egg}\text{length}}X100$$

#### Egg breaking force

Egg breaking force was measured by subjecting the eggs to compression pressure on an Instron 6022 machine (Instron, UK). The eggs were compressed along the x-axis at a constant speed of 20mm/min by a 100 kg load cell, and the crosshead lowered to compress the eggshell. The maximum force required to crack each egg was extracted from the Instron software (Blue Hill Universal)

#### Yolk index

The egg was broken onto a flat surface, and the yolk height and width were measured with a digital caliper (Carbon Fiber Composite ± 0.2 mm accuracy). The yolk index was then calculated by dividing the yolk height by the yolk width.

$$\text{Yolk}\text{index}=\frac{\text{Yolk}\text{height}}{\text{Yolk}\text{width}}X100$$

#### Haugh unit

Albumen height was first measured using a digital caliper. Then, the Haugh unit was calculated as 100log (H-1.7w^0.37+7.6) where H is albumen height (mm)

W is egg weight (g)

100, 0.37, and 7.6 are constants (Wei et al., 2020)

#### Egg surface area

The egg surface area was calculated according to Sezer (2007)

Egg surface area = 3.9782W^0.7056^, where *W* = egg weight (g) and 3.9782 and 0.7056 are constants.

#### Eggshell index

The eggshell index was calculated using the Ahmed et al. (2005) formula as;

$$\text{Eggshell}\text{index}=\frac{\text{shell}\text{weight}}{\text{egg}\text{surface}\text{area}}X100$$

#### Dry shell weight and eggshell thickness

Egg contents were emptied, and eggshells with the membranes were carefully rinsed under low-pressure water to get rid of all albumen. Precautions were taken to prevent shell loss, and shells were then air-dried for about 48 hours. Thereafter, the shell was weighed, and shell thickness was measured by using a digital micrometer screw gauge (Digital micrometer model 0-25 mm, 0.001 mm, China) to determine thickness from the pointed, narrow and middle part of the eggs. The average shell thickness was taken as the mean of the pointed, narrow, and middle points (Das et al., 2010).

### Hen euthanasia and keel dissection scoring

By week 70, 9 hens were lost to either injurious pecking or an unidentified cause. So, 111 hens were euthanized by a veterinarian using intravenous injection of 0.3 ml of Pentobarbital sodium. The hens were stored in the chiller for 24 hours and then dissected to score the keel bones visually. Keel fracture severity was classified by referring to a 3-D photographic model of a 4-point scale of keel severity as previously used by Toscano et al. (2015). Severity ranged from 0 to 3, depending on the presence and size of callus (described as minimal or pronounced, Thøfner et al., 2020) and fracture gaps on the dorsal or ventral surfaces of the bone. Based on the score, 21 hens were assigned Score 0 (no fracture), 25 hens were assigned score 1 (slight fracture having 1 fracture site and minimal callus formation), 29 hens were assigned score 2 (moderate fracture having 2-3 fracture sites and pronounced callus formation) and 36 hens were assigned score 3 (severe fracture having more than 3 fracture sites, pronounced callus formation and damage on the ventral, dorsal or lateral surfaces). Two birds were considered to have a fresh fracture due to an obviously new fracture line and hemorrhage, which must have occurred immediately after euthanasia. The new fracture was ignored in both hens, and scoring was only based on the old fracture. The severity scores were thereafter used to group the eggs previously collected for egg quality tests and incubation (Table 1). The score was also used subsequently to group the chicks that emanated from each hen.

**Table 1:** Number of eggs tested for egg quality and incubated according to their hens’ keel fracture severity.

Hen fracture status	Egg quality (1 day/batch)	Incubation (4 days/batch)
Batch 1	94	332
No fracture	16	66
Slight fracture	21	72
Moderate fracture	24	91
Severe fracture	33	103
Batch 2	77	396
No fracture	13	74
Slight fracture	16	84
Moderate fracture	24	105
Severe fracture	24	133
Batch 3	84	390
No fracture	16	69
Slight fracture	16	80
Moderate fracture	24	107
Severe fracture	28	134

### Chick housing and management

Across the three hatch batches, a total of 72, 63, 85, and 106 chicks emanated from 111 hens categorized as no fracture, slight fracture, moderate fracture, and severe fracture, respectively. The chicks were assigned to 4 different groups based on the keel fracture severity of their mother, i.e. group 0 (chicks that emanated from hens with no keel fracture), group 1 (chicks from hens with slight fracture), group 2 (chicks from hens with moderate fracture), and group 3 (chicks from hens with severe keel fracture). Chicks from each batch were weighed and tagged using the same color bands as their mother. A maximum of four chicks/hen were obtained in one batch; in this event, three of the chicks were further marked with a spot of green, blue, or red non-toxic markers on their head. Chicks from each batch were housed in two rooms, and each room was partitioned into two pens using a wire mesh and a cardboard covering 30 cm high to prevent visual contact; thus, within each batch, chicks from each of the four maternal fracture groups were kept separate. In total, three batches were housed across six rooms, with 12 groups of chicks. Each pen had the same resources, which included two heat lamps, two feeding troughs, and two drinkers. The chicks were grouped by their maternal keel fracture severity, i.e. one partitioned room housed groups 0 and 3 chicks, while the other partitioned room housed groups 1 and 2 chicks. Chicks were vaccinated against Newcastle disease on day 2. They were brooded for two weeks, with the temperature starting at 31 - 32 °c in the first week, reducing gradually to approximately 28 −30°C at week two, when the heat lamps were turned off. The chicks were provided ad libitum access to feed and water. The lighting regime was according to Bovans brown guidelines for commercial chicks (Bovans Brown Management Guide UK, 2022).

### Chick growth assessment (week 1-5)

The body weight of individual chicks was measured at hatch (day 0) and once weekly until week five. Total weight gain was calculated as the difference between body weight at week five and day 0. The total weight gain was divided by 35 days (five weeks) to obtain the average daily weight gain.

### Fear tests

At weeks four and five, respectively, individual tonic immobility and novel arena tests were carried out. All tests were done using two identical rooms and test apparatuses. To prevent flight reaction and ease stress during capturing, the lights in each room were dimmed to 4 lux before catching. Individual chicks were then gently caught with both hands and taken to the test rooms, which had lights at 20 lux.

#### Tonic immobility test

The tonic immobility (TI) test was conducted using the procedure described by Janczak *et al*. (2007). Each chick was placed on its back in a V-shaped wooden cradle while gently placing hands over its head and legs. The chicks were held in this position for 20 s, after which the hands were slowly removed from the birds. If the chicks remained motionless for 10 s after release, they were deemed to have entered tonic immobility, and the duration of TI was recorded for each chick using a stopwatch. However, when the chick self-righted within 10 s of release, the procedure was repeated, up to three times, and the number of induction attempts was recorded. Chicks that failed to enter TI after three attempts were scored as “no TI induced”, and were given a righting time of 0 s. The maximum duration of TI was set at 180 s, at which point the birds were encouraged to self-right and removed from the cradle.

#### Novel arena test

Each bird was immediately placed in the center of a novel, barren room (3.41 m x 4.4 m x 2.15 m LxWxH) between two novel objects (a dumbbell and a rubber lattice ball) that were 30 cm apart. The bird’s behavior was recorded for 5 minutes by an overhead camera. The video was thereafter observed in Boris (Friard and Gamba, 2016), and data were extracted on the latency to the first movement, number of defecations, duration of freezing, duration of walking and standing alert, number of novel object pecks, and the number of escape attempts. The ethogram for the behavioral observations is described in Table 2.

**Table 2:** Ethogram of chicks' behavior during the novel arena test.

Behavior code	Description
Escape attempt	Bird attempts to jump and/or fly out of the test arena
Fly	Wing flapping resulting in movement of the bird above the ground and around the center of the test arena
Latency to first movement	Time from when the bird is placed to when it takes its first step
Object peck	Bird directs its beak and pecks at any part of the novel object
Run	Bird takes hurried steps in the test arena
Sit alert	Bird sits with an alert body, an upright neck, and head
Sit inactive	Bird remains still in a sitting position, with eyes partially or fully closed.
Stand alert	Bird stands with an alert body, an upright neck, and head
Freeze	Bird appears still while standing with or without its head lowered
Walk	Bird makes unhurried steps in the test arena

The ethogram was constructed with reference to Nicol et al. (2009)’s definition.

### Statistical analyses

Using R 4.5.0, all data were initially subjected to normality tests. Data on egg quality, fertility, hatchability, chick weight, latency to first move, durations of standing alert and walking were analyzed using a Linear Mixed Model (LMM) with keel fracture severity (4-point score) as a fixed factor, batch and rooms as random factors, as appropriate. Additionally, for chick weight and behaviour data, chick room was nested within batch as a random factor. Logistic regression models were used to analyze differences in the likelihood of freezing, flying, sitting inactive, and sitting alert between fracture groups, while ordinal regression models were used to analyze differences in the likelihood of defecation, running, escape attempt, object pecking, and tonic immobility attempt between fracture groups. Significant means were separated using Bonferroni due to multiple comparisons. Correlation between egg quality, egg breaking force, and reproductive traits was tested by using Pearson correlations. The confidence interval was at 95 %.

## Results

### Keel fracture severity and egg quality

Means of the egg quality measures and the results of LMMs are summarized in Table 3. In LMMs, including room and batch as random factors, there was a significant difference between keel fracture groups for dry shell weight (*F* = 10.260, *P* < 0.001), eggshell thickness (*F* = 93.874, *P* < 0.001), eggshell index (*F* = 6.988, *P* < 0.001) and egg breaking force (*F* = 12.045, *P* < 0.001). Bonferroni mean comparison showed that the dry shell weight was lower in hens that sustained moderate (*P* = 0.007) and severe (*P* < 0.001) keel fractures compared with those without fracture. Within the three fractured groups, the dry shell weights of severely fractured hens were lower (*P* = 0.025) than the slightly fractured hens. Bonferroni mean comparisons showed that hens from the three fractured groups laid eggs with thinner shells (*P* < 0.001) compared with those without fracture. Within the three fractured groups, shell thickness decreased as fracture severity increased, whereby slightly fractured hens laid eggs with thicker shells compared with moderately fractured hens (*P* = 0.015), and eggs from the severely fractured hens had the lowest shell thickness (*P* < 0.001). Bonferroni mean comparison showed a lower eggshell index in hens with severe fractures (*P* < 0.001) compared with those without fractures. Within the three fractured groups, there was no difference (*P* > 0.05) in the eggshell index of slightly, moderately, and severely fractured hens. Bonferroni mean comparisons showed that the three fractured groups laid eggs with lower egg-breaking force (*P* = 0.001) compared with those without fracture. Within the three fractured groups, there was no difference in egg-breaking force between the slightly, moderately, and severely fractured hens. There was no difference (*P* > 0.05) between keel fracture severity groups for other measures of egg quality, such as egg weight, length, width, yolk height, average yolk width, albumen height, egg shape, yolk index, Haugh unit, and egg surface area.

**Table 3:** Mean (and standard error, SE) of egg quality outcome by the keel fracture severity.

Egg quality	No fracture *n* = 43	Slight fracture *n* = 48	Moderate fracture *n* = 72	Severe fracture *n* = 79	P-value
Egg weight (g)	61.88 (0.75)	62.21 (0.71)	61.00 (0.60)	60.30 (0.58)	0.092
Egg length (mm)	55.53 (0.44)	56.00 (0.43)	55.11 (0.39)	55.26 (0.39)	0.123
Egg width (mm)	44.18 (0.30)	44.09 (0.29)	44.39 (0.29)	44.00 (0.24)	0.467
Yolk height (mm)	20.94 (0.47)	21.18 (0.46)	20.83 (0.45)	20.72 (0.45)	0.201
Average yolk width (mm)	41.35 (0.32)	41.39 (0.30)	41.11 (0.26)	40.97 (0.26)	0.514
Albumen height (mm)	11.02 (0.46)	11.93 (0.45)	11.49 (0.43)	11.50 (0.43)	0.102
Dry shell weight (g)	5.38 (0.12)^a^	5.12 (0.12)^ab^	5.03 (0.11)^bc^	4.82 (0.11)^c^	<0.001
Shell thickness (mm)	0.41 (0.006)^a^	0.37 (0.006)^b^	0.35 (0.005)^c^	0.31 (0.005)^d^	<0.001
Eggshell index	7.37 (0.19)^a^	6.97 (0.19)^ab^	6.97 (0.18)^ab^	6.72 (0.17)^b^	<0.001
Egg shape index	79.68 (0.67)	78.85 (0.64)	80.56 (0.55)	79.66 (0.55)	0.103
Yolk index (%)	50.66 (1.16)	51.19 (1.14)	50.73 (1.10)	50.72 (1.10)	0.875
Haugh unit	102.95 (1.79)	106.39 (1.75)	104.74 (1.66)	104.93 (1.66)	0.119
Egg surface area (cm^3^)	73.04 (0.62)	73.31 (0.59)	72.30 (0.50)	71.72 (0.48)	0.089
Egg breaking force (N)	40.16 (1.55)^a^	32.63 (1.47)^b^	31.59 (1.25)^b^	30.15 (1.22)^b^	<0.001

P-values indicate associations between outcome and keel fracture severity (^abcd^ means with different superscript within a row differ significantly (*P* < 0.05).

### Keel fracture severity and reproductive traits

**Fig. 2:**
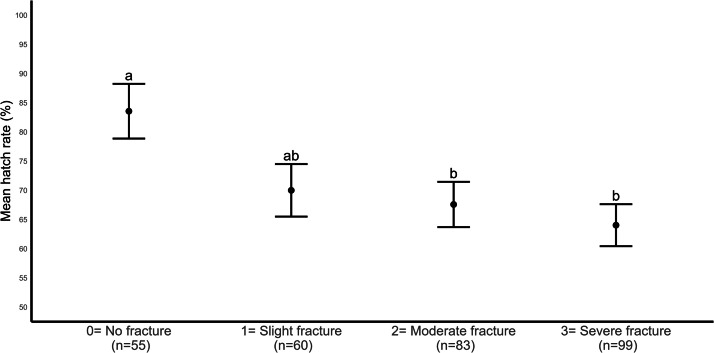
Mean hatch rate by keel fracture severity (^ab^ means with different superscripts differ significantly, *P* < 0.05).

Fig. 2 shows the mean hatch rate according to the hen’s keel fracture severity (with three incubation batches and room serving as random factors). The LMM showed a significant difference in hatch rate (*F* = 4.460, *P* = 0.004) between keel fracture severity groups. A significantly lower hatch rate was recorded for eggs from hens that sustained moderate (*P* = 0.038) and severe (*P* = 0.003) keel bone fractures, compared with those without fracture. Within the three fractured groups, there was no difference in the hatch rate between slightly, moderately, and severely fractured hens. There was no association between keel fracture severity and hen fertility (*P* > 0.05).

### Correlation between egg quality and reproductive traits in chickens

**Fig. 3:**
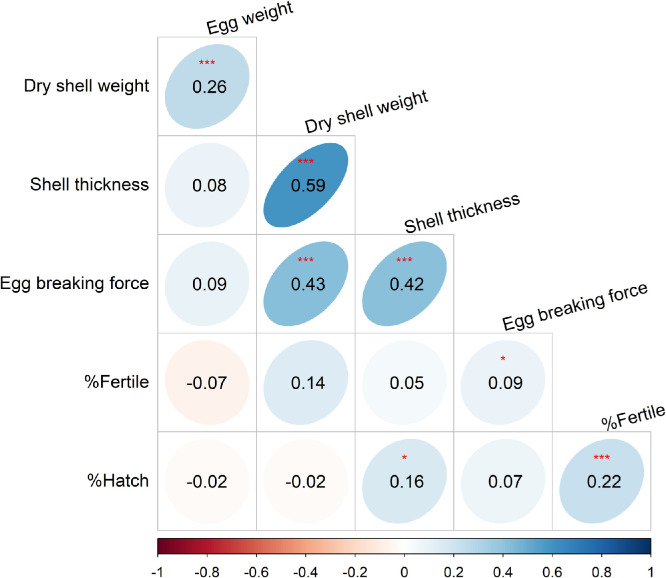
Pearson correlation matrix of egg quality and hen reproductive metrics. *P < 0.05, ***P < 0.001, required P-value = 0.003.

The relationship between some external egg qualities, egg breaking force, and hen reproductive traits was tested using Pearson correlations (Fig. 3). Bonferroni correction was done, and the required P-value for significance was *P* = 0.003. There were moderate positive correlations (*P* < 0.001) between egg breaking force, dry shell weight (*R* = 0.43), and shell thickness (*R* = 0.42). Between egg quality and reproductive traits, fertility correlated positively with egg breaking force; however, their correlation (*P* < 0.06) did not meet the required P-value for significance after Bonferroni correction. For hatch rate, a weak positive correlation (*P* < 0.05, *R* = 0.16) was found between shell thickness and hatch rate.

### Maternal keel fracture severity and chick growth

The mean growth metric of chicks grouped by maternal keel fracture severity is presented in Table 4. There was no difference (*P* > 0.05) in chick hatch weight, body weight at week five, total weight gain, and average daily weight gain between the four maternal keel fracture severity groups.

**Table 4:** Mean (and standard error, SE) of chick’s body weight by maternal keel fracture severity.

Chick quality	No fracture *n* = 67	Slight fracture *n* = 60	Moderate fracture *n* = 84	Severe fracture *n* = 105	P-value
Hatch weight (g)	41.51 (0.81)	42.24 (0.82)	40.83 (0.77)	40.82 (0.75)	0.086
Week 1 (g)	77.01 (2.78)	77.75 (2.80)	76.42 (2.75)	75.70 (2.73)	0.296
Final weight at week 5 (g)	404.68 (4.32)	406.31 (4.48)	401.77 (3.88)	404.49 (3.67)	0.840
Total weight gain at week 5 (g)	363.25 (4.30)	363.90 (4.46)	360.95 (3.86)	363.78 (3.65)	0.919
Average daily weight gain (g)	10.38 (0.12)	10.40 (0.13)	10.31 (0.11)	10.39 (0.10)	0.919

### Maternal keel fracture severity and chick behavior during novel arena tests

**Fig. 4:**
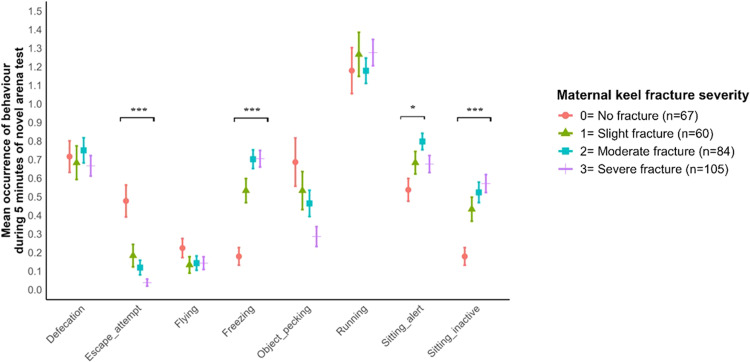
Mean frequency of occurrence of fear behaviors in chicks by maternal keel fracture severity (*= *P* < 0.05, *** = *P* < 0.001).

The frequency of occurrence of behavior in chicks from hens with different keel fracture severities during novel arena tests is presented in Fig. 4. Ordinal logistic regression model showed a significant likelihood of escape attempt occurring in relation to keel fracture (χ² (3) = 34.04, *P* < 0.001). Relative to chicks from fracture-free hens (group 0), chicks from slightly fractured, moderately fractured and severely fractured hens (groups 1-3) had lower odds of performing an escape attempt (OR = 0.28, *P* = 0.046; OR = 0.19, *P* = 0.002; OR = 0.07, *P* = 0.002, respectively). Within the three fractured groups, pairwise comparison of odds ratios shows no difference in the odds ratio of chicks performing escape attempts. Binary logistic regression models showed a significant difference in the likelihood of observing freezing behavior (χ² (3) = 56.30, *P* < 0.001). Relative to chicks from fracture-free hens (group 0), chicks from slightly fractured, moderately fractured and severely fractured hens (groups 1-3) were more likely to freeze (OR = 4.92, *P* = 0.002; OR = 10.40, *P* < 0.001; OR = 13.45, *P* < 0.001, respectively). Within the three fractured groups, pairwise comparison showed no difference in the odds ratio of observing freezing regardless of fracture severity. Binary logistic regression models showed a significant difference in the likelihood of observing sitting inactive (χ² (3) = 30.36, *P* < 0.001). Relative to chicks from fracture-free hens (group 0), chicks from moderate and severely fractured hens had a higher likelihood of sitting inactive (OR = 4.54, *P* = 0.001; OR = 7.02, *P* < 0.001, respectively). Within the fractured groups, pairwise comparison shows no difference in the odds ratio of sitting inactive regardless of fracture severity. Binary logistic regression models showed a significant difference in the likelihood of chicks sitting alert between keel fracture groups (χ²(3) = 9.77, *P* = 0.021). Relative to chicks from fracture-free hens (group 0), chicks from moderately fractured hens (group 2) had higher odds of sitting alert (OR= 3.24, *P* = 0.013). Within the fractured groups, there was no difference in the odds ratios of chicks sitting alert. There was no association between maternal keel fracture severity (*P* > 0.05) and the odds of observing defecation, object pecking, running, or flying. Similarly, the linear model showed no significant association (*P* > 0.05) between maternal keel fracture severity and latency to first move, durations of standing alert, or walking.

### Maternal keel fracture severity and chick response during tonic immobility test

The tonic immobility responses of chicks from hens with different keel fracture severities show no effect of maternal keel fracture severity group (*P* > 0.05) on the duration of tonic immobility and the odds ratio of tonic immobility attempt (*P* > 0.05).

## Discussion

This study aimed to examine the associations between the keel fracture severity of layer breeders and their egg quality, reproductive metrics, chick growth, and fear behaviors. Scoring of keel bones relied on dissection and visual examination of all keel bones to ensure accuracy. Based on the visual observations, the majority of the 70-week-old hens (88 out of 90) categorized with slight, moderate, and severe fractures in this study displayed healed keel fracture status as indicated by visible hard callus without fracture gap, inflammation, or haemorrhage. This is on par with studies that found healed and old fractures mostly in hens at the end of lay (Wilkins et al., 2004, 2011; Edgar et al., 2023).

Among the egg qualities that influence embryonic survival and hatchability are egg weight, eggshell quality, and the consistency of internal components such as yolk and albumen (Narushin and Romanov, 2002; King’ori, 2011), making them essential considerations for layer breeders. Likewise, the quality of an eggshell is measured by its weight, thickness, and mechanical strength (Tougan and Thewis, 2024). The current study found reduced dry shell weight, shell thickness, and shell index in eggs that emanated from keel-fractured chickens. Just as calcium is mobilized for shell formation from the medullary bone (Dacke et al., 1993; Kim et al., 2012), calcium is also required for fracture healing (Scholz et al., 2008; Einhorn and Gerstenfeld, 2015; Fischer et al., 2018). Previous reports of reduced shell thickness in keel-fractured chickens between early to peak production age (between 22 and 42 weeks) (Candelotto et al., 2017; Nasr et al., 2012a; Wei et al., 2020) suggest that the incidence of keel fracture initiated some calcium redirection to support bone healing at the expense of eggshell formation. On the other hand, it is possible that the hens had an inherent poor calcium metabolism, making them susceptible to fractures that resulted in lower eggshell quality. A study by Edgar et al. (2023) found that the incidence of healed fractures was associated with a reduced percentage of eggshell membrane but not shell thickness. The variation between this study and ours could be attributed to the fact that in the current study, the membrane was not removed, so it was measured along with the eggshell. Additionally, the variations may be a result of a more comprehensive scoring scale adopted in the current study, which accounted for degrees of fracture. Eggshell quality is a useful measure of calcium uptake efficiency and egg structural integrity (Candelotto et al., 2017), the poor shell quality in the current study may be linked to the possibility of depletion of calcium storage in fractured hens, as supported by research where the incidence of keel fracture correlated with lower bone mineral content and breaking strength (Nasr et al., 2012a; Tarlton et al., 2013; Toscano et al., 2013). The poor shell quality was further reflected in the lower egg-breaking force recorded in keel-fractured hens when subjected to compression force in this study. Breaking force is a measure of egg mechanical strength, and this has been positively correlated with shell thickness (Icken et al., 2006; Sun et al., 2012). According to Kim et al. (2012), hens producing soft-shell eggs possibly have poor medullary bone osteoclast cells that lack the ruffled borders that are essential for bone resorption, consequently limiting the supply of calcium for shell formation. The lower dry shell weight, shell thickness, eggshell index, and egg breaking force in 70-week-old hens with healed fractures may suggest that even after healing, there is a long-term disruption of medullary bone function, and the calcium distribution process towards egg formation remains impaired. However, as this study could not ascertain causality, it is unclear whether there was an underlying low calcium metabolism in the fractured hens that predisposed them to fracture, poor shell quality and egg breaking strength.

In addition to the differences between fractured and non-fractured hens, the shell quality results revealed that different degrees of fracture (i.e. sight, moderate, and severe) are associated with different eggshell parameters in some cases. Between the three fractured groups, shell index and breaking force remain similar irrespective of severity, suggesting that the reduction in these egg qualities was not amplified by additional fractures. This further implies that a slight incidence of fracture might be a threshold enough to compromise the shell index and breaking force, and it makes no further difference if a slightly fractured hen continues to sustain more fractures. This was, however, not the case for dry shell weight and shell thickness, which showed some further decline, especially with severely fractured hens laying eggs, which displayed the lowest dry shell weight and shell thickness. This result partially aligns with Candelotto et al. (2017), who recorded thinner eggshells, but with lower breaking strength as the likelihood of fracture increased in crossbred and pure line chickens at 26 weeks of age. Eggshell index, like shell thickness, is a measure of calcium carbonate quantity in an eggshell (Ahmed et al., 2005), and a lower shell index could indicate weaker eggshell strength with higher chances of cracks. While Wei et al. (2023) reported lower calcium, higher parathyroid and calcitonin in serum samples from fractured hens, the authors associated the lower calcium circulation with poor keel bone mineralization. They also associated the higher parathyroid with hypocalcemia inducement during the eggshell mineralization, to increase Ca concentration and bone absorption (Singh et al., 1986). The study also reported the upregulation of some collagens that enhance bone matrix synthesis and mineralization following fracture to stimulate calcium signaling pathways towards fracture healing. Considering that the study reported the serum concentration of minerals and molecules in fractured hens irrespective of the degree of fracture, possible variations in serum calcium by degree of fracture cannot be ascertained. However, the association between graded shell thickness and fracture severity in this current study suggests that severe fractures could interfere with calcium metabolism, possibly resulting in even poorer bone and progressively thinner shells. According to Casey-Trott et al. (2015), fracture healing initiates callus formation that demands high mineral content due to bone remodeling of the callus during the healing process. This may suggest that a higher number of and/or more severe fracture cases result in more calcium redirection, further depriving the shell formation process. The sustainability of the breeder industry lies in the ability of layer breeders to produce hatchable eggs with shells that can withstand incubation. Eggs with lower shell thickness and breaking strength are susceptible to cracks during handling and transportation, thereby reducing the proportion of eggs set for incubation. In an economic sense, loss from egg breakages may reduce hatchery efficiency, output, and return on the breeder flock.

In the current study, the absence of association between fracture severity, egg yolk and albumen qualities suggests that hens could sustain good egg size and internal quality despite skeletal issues, probably because of good dietary nutrition and efficient allocation of resources towards oocyte maturation and yolk development. These results align with Wei et al. (2020), who reported an effect of keel fracture on egg external qualities but not internal qualities. Also, reports from previous studies on egg size and internal components (albumen height, Haugh unit) suggest the likelihood of these egg parameters being predominantly influenced by age (Lukáš et al., 2009; Tůmová and Ledvinka, 2009; Barrett et al., 2019; Quan et al., 2021; Vlčková et al., 2019) and nutrition (Keshavarz et al., 2003; Roberts and Ball, 2004). The Haugh unit is the quality of egg protein as measured by albumen thickness, which is particularly influenced by changes in protein gel structure, as well as the content of ovalbumin, ovomucin, and lysozyme (Robert, 2004). This further suggests that the regulatory process for egg internal components may likely be more affected by metabolic stress rather than stress relating to keel bone fracture, or more specifically, healed fracture cases, as in the current study.

Fertility and hatchability are measures of reproductive quality and are quite sensitive to environmental factors (Babacanoğlu et al., 2013; Sas et al., 2006; Stromberg, 1975). Previous studies found that stress experienced by hens can influence oocyte development in chickens (Sas et al., 2006; Babacanoğlu et al., 2013) through the transfer of stress and reproductive hormones (Groothuis and Schwabl, 2008; Henriksen et al., 2011; Bertin et al., 2013). The current study found no association between keel fracture severity and egg fertility. While fertility could be a male factor (Parker et al., 2000; Fouad et al., 2020), especially when hens continuously lay a high number of eggs, hens could also contribute to fertility either through a reduction in sperm storage, a disrupted ovulation cycle that misses sperm viability windows or even poor oocyte quality which could potentially be caused by stress or hormonal disruption (Groothuis and Schwabl, 2008). A study by Nasr *et al*. (2012b) related a reduced egg production in keel-fractured hens to the possibility of pain and stress-induced delay in ovulation through the disruption of luteinizing hormone secretion and surge. Although this study could not measure egg production due to the individual egg collection procedure adopted, the result of this study indicates that any residual pain and stress after fracture healing may not be enough to alter the reproductive process relating to egg fertility. The current study found an association between hens’ fracture severity and hatch rate, with lower hatch rates recorded in moderate and severely fractured, but not in slightly fractured hens. These results may imply a threshold effect wherein an association between keel fracture and hatch rate only becomes pronounced when a hen continually sustains fractures. This is the first study to examine the association between fracture and reproductive output. Storage and incubation conditions can affect egg hatchability (King’ori, 2011; Hedlund and Jensen, 2021; Tona et al., 2022; Bilalissi et al., 2022), which is why precautions were taken to rule out the incubation effect by ensuring eggs from all hens and fracture groups were well mixed and fairly represented in all the hatching chambers. The explanation for the reduced hatch rate among fractured hens may be connected to the poor eggshell quality obtained in this study. Eggshell characteristics may influence embryonic development by interacting with the physiological features of developing embryos. Eggshells also influence heat transfer (Boleli et al., 2016), regulate moisture retention (Rahn et al., 1979), and offer microbial protection to the embryo (Sauter and Petersen, 1974). Consequently, eggs with thinner shells may lose excess water during incubation, lowering embryo viability, increasing dead-in-shell, and resulting in poor hatch rates. As a consequence, if a lower economic return could result from the low hatchability of eggs from fractured hens, this may contribute to the early depopulation of breeder flocks that would ordinarily be kept for some additional time.

This study found positive correlations between egg weight, shell weight, and shell thickness. The correlations between these physical egg qualities align with previous studies (Kim et al., 2005; Alfonso-Carrillo et al., 2021). The positive relationship between breaking force, shell weight, and thickness suggests that shell qualities are a good measure of egg mechanical strength. This is similar to what has been established in some previous research (Sun et al., 2012; Icken et al., 2006). Interestingly, hatch rate had a weak positive correlation with shell thickness. A similar positive correlation has been previously reported where eggs with thicker shells had higher hatchability (Liao et al., 2013). Given the recorded negative association between keel fracture and most shell qualities and hatch rate, one would have expected a stronger correlation between the shell qualities and the hatch rate. However, critical evaluation of the pairwise comparison between the different fracture severities, particularly for hatch rate and shell thickness, reflects the weak relationship. For instance, when the shell thickness was reduced with increasing fracture severity, the reduction did not directly correspond to reduced hatch rate until the fracture became moderate and severe. This suggests that small variations in egg quality, such as shell weight and thickness, do not result in reduced hatch rate; however, moderate and severe fractures appear to result in pronounced reduction in shell thickness with an associated reduction in hatchability. In addition, the weak correlation between shell thickness and hatch rate confirmed that a moderately thick shell is required for good hatchability (Narushin and Romanov, 2002; King’ori, 2011). According to Icken et al. (2006), the normal range for shell thickness is from 0.35 to 0.40 mm. Lower thickness resulted in lower hatchability (Bennett, 1992). On the other hand, thicker shells may affect moisture exchange, causing early embryonic mortality (Peebles and Brake, 1985). Excessive thickness may hinder chick piping during hatching, which could result in delayed hatching or embryonic mortality.

Previous studies have established some direct relationships between maternal stress, chicks’ brain function (Tomasi, 2024), growth performance (Hynd et al., 2016; Bowling et al., 2018; Angove and Forder, 2020), and behavior (Janczak et al., 2007; Ericsson et al., 2016). This study examined whether there was any association between maternal keel fracture severity and chick growth performance. Contrary to our hypothesis, there was no association between maternal keel fracture and chick growth up to five weeks of age. Considering that the hens in this study had healed fracture cases, healed fractures may exhibit absent/lesser pain, and therefore any associated stress may not be sufficient to elicit an effect on the growth pattern of the offspring chicks. The susceptibility of hens to new keel fractures improves after about 49 weeks (Stratmann et al., 2015; Toscano et al., 2018; Rufener et al., 2019). To conclude whether maternal fracture affects chick offspring, it would be worth correlating maternal fracture and chick growth during the time window when hens are more prone to fresh or healing fractures.

This study used tonic immobility and novel arena tests to measure fear in chicks from hens with different keel fracture severities. Chicks that emanated from keel-fractured hens demonstrated lower escape attempts, higher occurrence of freezing, and sitting inactive, which are not severity dependent. Escape and freezing behaviors have been respectively categorized as active and passive behavioral responses to threat (Jones, 1996; Erhard and Mendl, 1997, 1999). The result of this study suggests that maternal fracture altered fear behavioral patterns in chicks, causing a shift from active to passive responses in the novel arena. According to Forkman *et al*. (2007), lower escape attempts either imply being more afraid or animals having less motivation for social reinstatement. This thereby indicates that the chicks from fractured hens, irrespective of severity, had more passive behavioral responses to fear and with less motivation for conspecific reinstatement. Fear during early life has been correlated with high stress sensitivity (de Haas et al., 2012) and the development of feather pecking in later life (Rodenburg et al., 2004). Further research could explore the implications associated with these behavioral changes on the welfare and production of the chicks at their laying phase.

This study acknowledges some limitations that could affect the interpretation and the conclusions. The first is the variation in sample size between fracture severities, which could not be controlled at the beginning due to the reliance on the hen dissection score that was only obtainable at the end of data collection, when hens were euthanized. We adopted the Bonferroni adjustment to help correct some bias that may result from unequal sample sizes. The second limitation was the room grouping of chicks during the study, where chicks were grouped by maternal keel fracture severity into two partitioned rooms. Two groups of chicks from each batch were in one room, while the other was in the second; this could potentially introduce some room effect, which was accounted for in the statistical analysis. Future studies could aim to control for room effects more efficiently by randomizing chicks regardless of maternal fracture severity across rooms. The nature of this study involved collecting hens after keel fracture had occurred and likely healed, which means this study could not deduce the causality of the effect seen on both eggs and chicks. There may be some individual differences present before hens sustained a fracture, which could also cause or be associated with the fracture, i.e. increased fearfulness, which could also affect the occurrence of keel fracture, egg quality, or chick behavior.

## Conclusions

This study found lower dry shell weight, shell index, and egg-breaking force in eggs from fractured hens compared with those without fractures. Within the three fractured groups, there were no differences in shell index and egg breaking force irrespective of severity. We concluded that a slight fracture might be a threshold enough to compromise the shell index and egg-breaking force. Dry shell weight, shell thickness, and hatch rate of breeder hens were lowest among moderate and severely fractured hens. At higher prevalence, this may have implications for the managerial practice and economic return of a breeder flock. Maternal fracture was not associated with chick body weight but was associated with a shift in the fear behavioral pattern of chicks, from active to passive responses in a novel arena. Further research should consider associating chick growth with keel fracture during the time window when hens have a higher likelihood of fresh fracture, as well as the implications of the fear-related behavioral changes on the welfare and production of the chicks at their laying phase.

**DATA AVAILABILITY**

The data would be available on the University of Bristol Research Data Repository.

**DISCLOSURES**

There is no conflict of interest to disclose

## CRediT authorship contribution statement

**M.O. Logunleko:** Writing – review & editing, Writing – original draft, Visualization, Software, Resources, Project administration, Methodology, Investigation, Formal analysis, Data curation, Conceptualization. **S.L. Lambton:** Writing – review & editing, Supervision, Project administration, Methodology, Investigation, Funding acquisition, Conceptualization. **G.J. Richards:** Writing – review & editing, Resources, Methodology, Investigation, Formal analysis. **J.L. Edgar:** Writing – review & editing, Supervision, Resources, Project administration, Methodology, Funding acquisition, Conceptualization.

## Disclosures

The authors declare that they have no known competing financial interests or personal relationships that could have appeared to influence the work reported in this paper.
